# First person – Carly York

**DOI:** 10.1242/bio.057109

**Published:** 2020-11-05

## Abstract

First Person is a series of interviews with the first authors of a selection of papers published in Biology Open, helping early-career researchers promote themselves alongside their papers. Carly York is first author on ‘Squids use multiple escape jet patterns throughout ontogeny’, published in BiO. Carly conducted the research described in this article while a PhD candidate in Ian Bartol's lab at Old Dominion University, Norfolk, USA. She is now an Assistant Professor of Biology at Lenoir-Rhyne University, investigating areas of animal physiology and biomechanics.


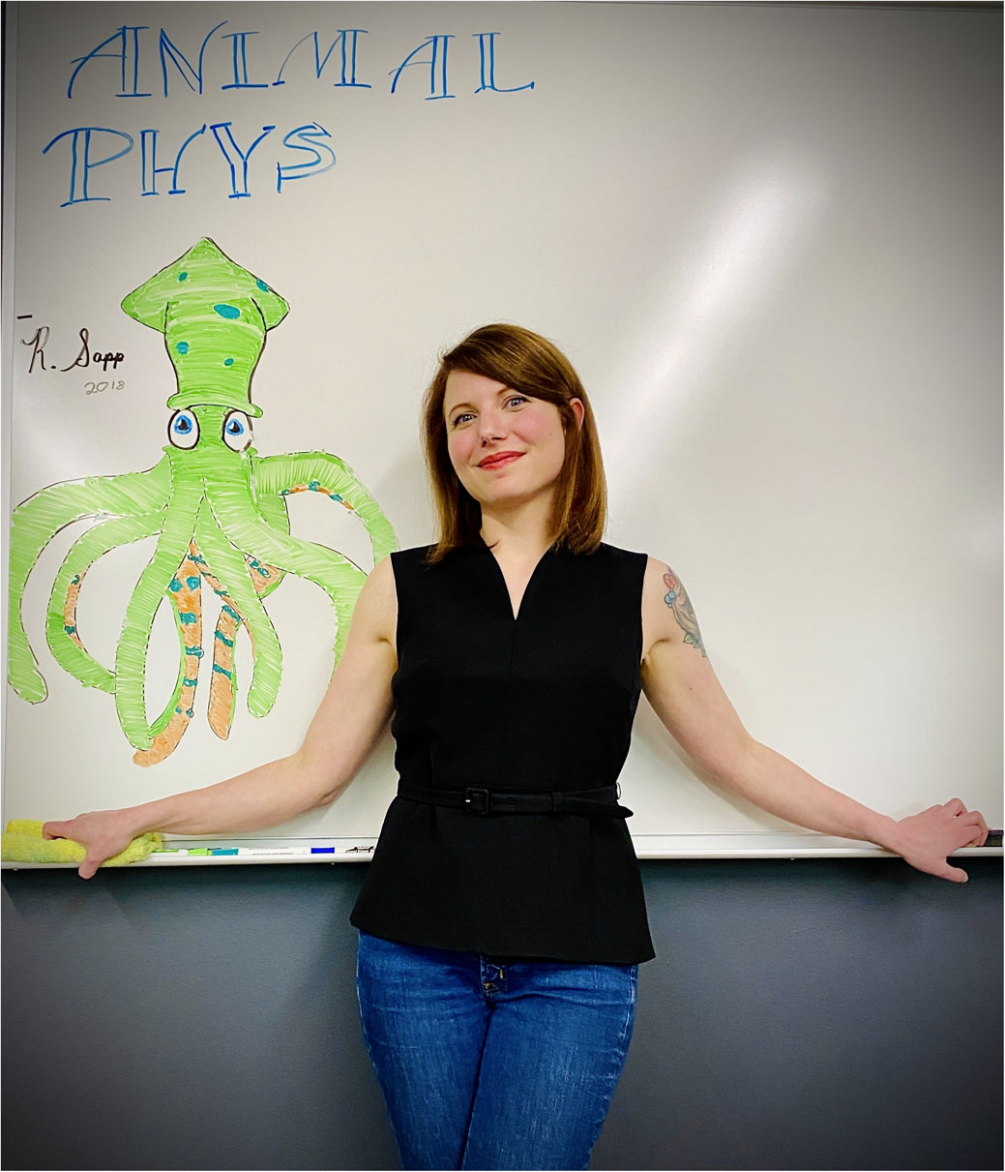


**Carly York**

**What is your scientific background and the general focus of your lab?**

I am an animal physiologist who has studied a number of different organisms including equines, cephalopods and amphibians. My master's degree research, which I completed at Western Kentucky University under the supervision of Dr. Bruce Schulte, focused on equine social behavior and stress physiology. I then completed my doctorate at Old Dominion University with Dr. Ian Bartol, where I investigated how squid evade predators using an array of sensory modalities and a jet propulsive escape response. I am currently an Assistant Professor of Biology at Lenoir-Rhyne University, where I study the sensory physiology of the African Clawed Frog (*Xenopus laevis*) to better understand their ecological impact as an invasive species. While my scientific path has been far from linear, I have gained invaluable skills by working in different animal systems.

**How would you explain the main findings of your paper to non-scientific family and friends?**

Squid belong to a class of animals called the cephalopods, which also includes the octopus, cuttlefish and nautilus. These animals have a fossil record dating back 500 million years, indicating that this class has been an evolutionary success, despite having numerous predators, such as fish and marine mammals. Squid have a unique way of swimming called jet propulsion. Unlike a fish that undulates its fins back and forth to propel itself forward, a squid pulls water into its body and pushes it out of a small funnel. This push of water thrusts the animal into an escape jet, allowing it to quickly escape a predator. We used to think that this way of swimming was inefficient because the animal would need to stop and refill its body, but by measuring how the water moves as it leaves the squid's body, we now know that it is actually a very efficient mode of locomotion. It is likely that this efficient, and effective, mode of escape allows squid to successfully evade their many predators.

**What has surprised you the most while conducting your research?**

As a doctoral student, you often underestimate the amount of luck required for successful data collection. This project required a tremendous amount of perseverance from our team. At the time that this research was conducted, we were relatively new to working with the three-dimensional particle imaging velocimetry (PIV) imaging system DDPTV (Defocusing Digital Particle Tracking Velocimetry). In addition to using new technology, we were working with squid, which are notoriously uncooperative animals. Waiting for a squid to compliantly swim in a water tunnel while a laser beam shines on them requires extreme patience.

In terms of our results, we were surprised by the high efficiencies that we calculated in these escape jets throughout paralarval, juvenile and adult life stages. Historically, jet propulsion at high velocities has been considered to have low efficiency compared to caudal fin propulsion typically found in fish. However, our findings indicate that jet propulsion is a high-velocity, propulsively efficient escape mechanism throughout ontogeny in squid. We were also surprised to learn that squid have flexibility in escape responses, which was evident by the observation of two different escape jet modes throughout ontogeny. Escape jet I is more efficient in juveniles and adults and may be the mode used when a threat is not eminent. Escape jet II is less efficient than escape jet I and may be used when a predatory attack is unavoidable, making a rapid escape integral for survival.

“As a doctoral student, you often underestimate the amount of luck required for successful data collection.”

**What, in your opinion, are some of the greatest achievements in your field and how has this influenced your research?**

The development of PIV was one of the greatest technological advancements for studying squid biomechanics. PIV is an optical measurement technique that allows for the measurement of the velocity and vorticity fields within a region of flow. By evaluating the 3D flow features of a squid's escape jet, we can visualize the vortices produced by this behavior, and calculate the efficiency in a new way.

**Figure:**
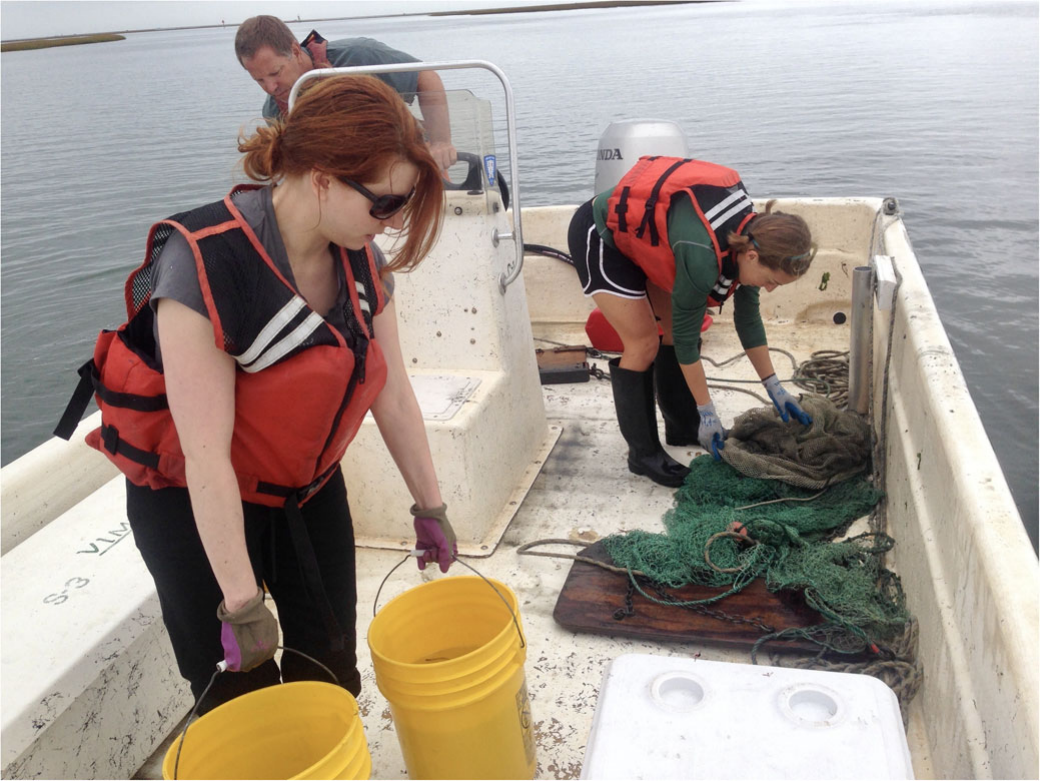
**Trawling for squid on the eastern shore of Virginia through the VIMS Marine Center.**

**What's next for you?**

As an assistant professor at a primarily undergraduate institution, my goals are to maintain a research program that allows for students to get hands on experience in the lab. I am currently working with a colony of African Clawed Frogs (*X. laevis*) and investigating their sensory physiology. I am particularly interested in focusing on how the lateral line system contributes to prey acquisition throughout ontogeny, and how their sensory physiology plays a role in the ecology of these animals as invasive species.
